# Abnormalities of white matter integrity in the corpus callosum of adolescents with PTSD after childhood sexual abuse: a DTI study

**DOI:** 10.1007/s00787-015-0805-2

**Published:** 2015-12-23

**Authors:** Mirjam A. W. Rinne-Albers, Steven J. A. van der Werff, Marie-José van Hoof, Natasja D. van Lang, Francien Lamers-Winkelman, Serge A. Rombouts, Robert R. J. M. Vermeiren, Nic J. A. van der Wee

**Affiliations:** 1 Curium-LUMC, Academic Center for Child and Adolescent Psychiatry, Oegstgeest, The Netherlands; 2 Department of Psychiatry, Leiden University Medical Center (LUMC), Leiden, The Netherlands; 3 Leiden Institute for Brain and Cognition (LIBC), Leiden, The Netherlands; 4 Psychotraumacenter and Department of Child and Adolescent Psychiatry, GGZ Rivierduinen, Leiden, The Netherlands; 5 Institute of Psychology and Education, Vrije Universiteit, Amsterdam, The Netherlands; 6 Department of Radiology, Leiden University Medical Center (LUMC), Leiden, The Netherlands; 7 Institute of Psychology, Leiden University, Leiden, The Netherlands

**Keywords:** PTSD, Diffusion tensor imaging, Adolescents, Sexual abuse, Neuroimaging

## Abstract

This study seeks to determine whether white matter integrity in the brain differs between adolescents with post-traumatic stress disorder (PTSD) due to childhood sexual abuse (CSA) and matched healthy adolescents and whether there is a relationship between white matter integrity and symptom severity in the patient group. Using 3T diffusion tensor imaging, we examined fractional anisotropy (FA) in a group of adolescents with CSA-related PTSD (*n* = 20) and matched healthy controls (*n* = 20), in a region of interest consisting of the bilateral uncinate fasciculus (UF), the genu, splenium and body of the corpus callosum (CC), and the bilateral cingulum. In addition, we performed an exploratory whole brain analysis. Trauma symptomatology was measured with the Trauma Symptom Checklist for Children (TSCC) to enable correlational analyses between FA differences and trauma symptomatology. The PTSD group had significantly lower FA values in the genu, midbody and splenium of the CC in comparison with controls (*p* < 0.05, tfce corrected). Post hoc analyses of the eigenvalues of the DTI scan showed increased radial and mean diffusivity in the patient group. In addition, we found a significant negative correlation between scores on the anger subscale of the TSCC and FA values in the left body of the CC in patients (*p* < 0.05). Adolescents with CSA-related PTSD show decreased FA in the CC, with abnormalities in the integrity of the left body of the CC being related to anger symptoms. These findings suggest that early trauma exposure affects the development of the CC, which may play a role in the pathophysiology of PTSD in adolescents.

## Introduction

Childhood psychotrauma is a prevalent and important predictor of both child and adult psychopathology as well as a number of somatic disorders [1, 26, 31, 45]. Preclinical research in rodents and non-human primates has shown that structure and functioning of the developing brain are highly vulnerable to the effects of adversity, especially in certain critical time windows. In animal studies, childhood adversity was found to be associated with changes in brain circuitry involved in stress and emotion regulation, such as the hippocampus and certain prefrontal regions, possibly underlying vulnerability to the impact of stressors later in life [12, 25, 32, 41]. In adult humans, a history of chronic traumatization during childhood and adolescence was found to be associated with structural and functional damage in key elements of emotion and stress regulating brain circuitry, for example in the hippocampus in adults reporting childhood abuse or in the medial prefrontal cortex in adults reporting childhood emotional maltreatment [5, 56]. Bearing in mind the neuroplasticity of the maturing human brain [8], a thorough understanding of the human neurobiology underlying the psychological sequelae of childhood and youth psychotrauma may hold promise for developing appropriate interventions to alter adverse neurodevelopmental trajectories [22]. Some recent reviews and meta-analyses have addressed structural brain alterations following childhood trauma in both adolescents and (young) adults with and without psychopathology [11, 40, 60]. The studies included in these reviews and meta-analyses often used different approaches and varied in sample size. Nevertheless, next to findings in the cerebellum and sensory cortex, most of the results from reviews and meta-analyses point toward involvement of the corpus callosum (CC) and corticolimbic circuits in the pathophysiological sequelae of psychotrauma in children and young adults.

Diminished white matter (WM) integrity can constitute one element of abnormalities in corticolimbic and related circuitry. Diminished structural integrity may impede adaptive emotional and cognitive functioning, thereby rendering an individual vulnerable to childhood and adult psychopathology.

A promising tool for examining the structural integrity of WM in children and youth who experienced psychotrauma is diffusion tensor imaging (DTI). Fractional anisotropy (FA) is the most commonly used DTI parameter and reflects the degree of diffusion directionality of water, which in white matter can be influenced by structural properties such as axonal density, organization and myelinization. Smaller FA values are associated with decreased white matter integrity. To further interpret differences in FA, additional parameters such as the mean diffusivity (MD), axial diffusivity (AD), and radial diffusivity (RD) can be assessed as well.

So far, four studies in children and youth have employed DTI to examine the effects of psychotrauma on white matter integrity in the developing brain and several reported abnormalities in the CC, but also in other areas. The first small study, in children who had been subjected to early socioemotional deprivation (*N* = 7), found decreased FA in the left UF [14]. The second study, in a group of children (*N* = 17) with post-traumatic stress disorder (PTSD) following varying forms of maltreatment, found reduced FA in the medial and posterior subregions of the CC [24]. The third study looked at the influence of Early Life Stress (ELS) on FA of the genu of the CC across the life span in healthy individuals. The results showed a lower FA in the youngest (8–12 years) and oldest (51–72 years) ELS age groups compared to non-exposed controls, suggesting that the effect is independent from the presence of psychopathology [44]. This was corroborated in the fourth study by Huang and Rao, in adolescents exposed to childhood maltreatment but without a history of psychiatric illness, who showed decreased FA values compared to controls in the left and right superior longitudinal fasciculi, right cingulum bundle, left inferior fronto-occipital fasciculus and splenium of the CC [21]. None of these studies investigated additional DTI parameters and only a region-of-interest (ROI) approach was used, which may have led important alterations in WM microstructure outside the ROI to go unobserved. Furthermore, study populations were heterogeneous for type of child adversity, which might have resulted in heterogeneous neuroimaging findings and differences in psychopathological sequelae.

The aim of our study was to investigate white matter integrity in a group of adolescents with psychopathology related to childhood sexual abuse (CSA) and matched healthy controls. We chose CSA as this is a prevalent form of child psychotrauma and a frequent cause of PTSD [15, 27]. This study is the first to focus on integrity of white matter tracts in a group of adolescents who had all experienced CSA. Based on previous neuroanatomical studies in children and youth as well as in adults, we hypothesized a reduced FA in the CC, the UF and the cingulum, although findings have not been unequivocal. We also aimed to investigate the possible relationship between FA and clinical symptoms in the patient group. Next, we also planned an exploratory whole brain analysis to detect aberrant FA values in areas outside our a priori defined ROIs.

## Methods

### Participants

We included *N* = 22 adolescents with a history of CSA and related PTSD (further described as PTSD group) and *N* = 30 healthy controls. The current cross-sectional study is part of the Emotional Pathways’ Imaging Study in Clinical Adolescents (EPISCA), a longitudinal MRI study in which adolescents are followed over a 6-month period.

Inclusion criteria for the patient group were having experienced sexual abuse during their lifetime more than once by one or more perpetrators in- or outside the family and being referred to a mental health service. Most participants came from specialized psychotrauma centers and had experienced severe and frequent sexual abuse. Presence of PTSD was not an inclusion criterion, but clinical assessments (see below) showed that all patients but one were having PTSD related to the CSA. Exclusion criteria were: (1) primary DSM-IV diagnosis of ADHD, pervasive developmental disorders, Tourette’s syndrome, obsessive–compulsive disorder, bipolar disorder, and psychotic disorders, (2) current use of psychotropic medication other than stable use of SSRI’s, or amphetamine medication on the day of scanning, and (3) current substance abuse. The healthy control adolescents were recruited through local advertisement, with the following inclusion criteria: no clinical scores on validated mood and behavioral questionnaires, no history of traumatic experiences and no current psychotherapeutic intervention of any kind. All participants met the following inclusion criteria: aged between 12 and 21, estimated full scale IQ (FIQ) ≥80 as measured by Dutch versions of the Wechsler Intelligence Scales for Children (WISC-III) [59] or adults (WAIS) [58], being right-handed, normal or corrected-to-normal vision, sufficient understanding of the Dutch language, no history of neurological impairments and no contraindications for MRI testing (e.g. braces, metal implants or possible pregnancy). More extensive description of the clinical group can be found in an earlier report about the EPISCA project [57].

The medical ethics committee of the Leiden University Medical Centre approved the study. All anatomical scans were reviewed and cleared by a radiologist. Written informed consent was obtained from all adolescents and their parents. Participants received a financial compensation including travel expenses.

### Clinical assessments

A standardized set of instruments was used to assess symptomatology in both groups of adolescents.

The Anxiety Disorders Interview Schedule Child and Parent Versions (ADIS-C/P) [46] are semi-structured interviews for the classification of DSM-IV anxiety and depressive disorders in children. The adolescents and at least one of their parents were interviewed. A minimal interference score of 4, obtained by trained examiners based on the ADIS-C and ADIS-P, is necessary for classification. The ADIS is known to have good reliability and validity [47] with reported strong test–retest reliability statistics for the ADIS-C/P for combined diagnoses (0.80–0.92) and individual diagnoses (0.62–0.88).

As brain development is known to be influenced by sexual development, physical sexual development was measured with the self-report Puberty Development Scale (PDS) [38]. The PDS consists of five items that are measured on a five-point scale by the examiner: 1 = pre-pubertal, 2 = early pubertal, 3 = mid-pubertal, 4 = late pubertal, 5 = post-pubertal. The PDS is considered a valuable instrument determining pubertal stage [4, 19].

The Trauma Symptom Checklist for Children (TSCC) [7] which measures trauma-related symptoms is a 54-item self-report for children and adolescents aged 8 through 18, but is often used up to 21 years [3, 18]. On a four-point scale (never to almost all of the time), the adolescent indicates how often a thought, a feeling or a behavior occurs. The items are grouped into six clinical scales. The clinical scales are Anxiety (Anx), Depression (Dep), Post-traumatic Stress (Pts), Sexual Concerns (Sc), Dissociation (Dis) and Anger (Ang). The TSCC total score is used as the main measure on post-traumatic symptomatology. Cronbach’s alpha coefficients reported range from 0.77 to 0.89 for subscales and 0.84 for the total scale. The questionnaire has extensively been studied, which has confirmed its good psychometric qualities [29, 36].The internal consistency of the TSCC subscales varied between 0.85 and 0.94, except for the Sexual Concerns subscale that measured 0.68.

Six subscales from the Wechsler Intelligence scales scores (picture completion, similarities, picture concepts, arithmetic, block design and comprehension) were converted into FIQ estimates.

### Data acquisition and preprocessing

DTI data were collected using a Philips 3.0T Achieva MRI scanner (Philips Medical Systems, Best, The Netherlands) with an eight-channel SENSE (Sensitivity Encoding) head coil. A single-shot echo-planar imaging sequence was used with the following scan parameters: repetition time = 11,000 ms, echo time = 56 ms, flip angle = 90°, *b*-factor = 1000 s/mm^2^, voxel dimensions = 2.3 mm isotropic, number of slices = 73, and no slice gap. DTI data were acquired along 32 directions, together with a baseline image having no diffusion weighting (*b* = 0). Total scanning time was ~7.5 min. Collected DTI data were preprocessed and analyzed, using the Oxford Centre for Functional MRI of the Brain (FMRIB) software library (FSL; http://fsl.fmrib.ox.ac.uk/fsl/fslwiki/) [49] version 5.0.2. First, DTI data were corrected for distortion and motion artifacts, induced by eddy currents or by simple head motions, using affine registration of each diffusion weighted image to the *b* = 0 reference image. Next, non-brain tissue was removed, using the Brain Extraction Tool. Finally, to generate individual FA maps for each participant, the diffusion tensor model was fitted to each voxel, using FMRIB’s Diffusion Toolbox. Total brain volume, normalized for subject head size, was estimated using SIENAX [51], part of FSL.

### Tract-based spatial statistics

Tract-based spatial statistics (TBSS) [48] version 1.2 was used for voxelwise analysis of the preprocessed FA data. First, individual FA images were aligned to the FMRIB58_FA standard-space image, using nonlinear registration. Next, the mean FA image was generated and thinned to create a mean FA skeleton, which represents the centers of all tracts common to the entire group. The mean FA skeleton was then thresholded at a FA value of ≥0.4, to exclude peripheral tracts and minimize partial voluming. Finally, each participant’s aligned FA images were projected onto the mean FA skeleton and the resulting data were fed into voxelwise permutation-based analysis.

### Region-of-interest TBSS

To test for regional specific FA alterations, we implemented an ROI-based TBSS. A binary mask, encompassing the bilateral UF, the genu, splenium and body of the CC and the bilateral cingulum, was created as region of interest using the Johns Hopkins University (JHU) white matter atlas provided by FSL [34]. The uncinate fasciculus connects subcortical subregions of the limbic system, such as the hippocampus and the amygdala, with the medial prefrontal cortex. The corpus callosum is the largest white matter bundle in the brain and connects left and right cerebral hemispheres. It consists of three subregions, namely the splenium (posterior), the body (middle), and the genu (anterior). The cingulum bundle is situated superior to the corpus callosum, curving around the genu and splenium. It connects prefrontal and subcortical areas, with additional projections to the parietal lobe.

The mask was then applied to the mean FA skeleton, to include only voxels comprised in the mean FA skeleton. This confines the statistical analysis exclusively to voxels from the center of the tract, thereby minimizing anatomic inter-subject variability, registration errors, and partial voluming. The resulting study-specific ROI mask was used for voxelwise permutation-based ROI analysis.

### Statistical analysis of demographic and clinical data

We used analysis of variance (ANOVA) to compare the two groups on age, IQ and TSCC total score. Because not all TSCC subscales showed normal distribution we used non-parametric analysis (Mann–Whitney) for the comparison of the TSCC subscales between the two groups.

### MRI analysis

Using FSL’s Randomize tool, permutation-based inferences with Threshold-Free Cluster Enhancement (TFCE) were carried out for voxelwise analysis of FA data [50]. 5000 random permutations were generated to build up the null distribution of the cluster size statistic, while testing the following contrasts: (1) controls > PTSD, (2) controls < PTSD. PDS score, total brain volume, gender and FIQ (demeaned across groups) were included in the analysis as nuisance regressors to correct for between groups variances. The resulting statistical maps were corrected for multiple comparisons across space (*p* < 0.05) and the JHU White Matter and Juelich Histological atlases were used to label clusters with significant FA alterations. This step was first carried out using the ROI mask to test our specific hypotheses. Next, we ran this step a second time using a whole brain mask for our exploratory analysis.

### Post hoc analyses

The association between FA and symptom severity in the PTSD group was examined using a voxelwise correlation approach. A mask was created of the voxels that were found to differ significantly on FA based on the between-group ROI analysis.

The TSCC total and subscale scores of the PTSD group were fed into FSL’s Randomize tool along with the mask, using permutation-based inferences with TFCE.

Last, we examined how the between-group differences in FA values related to the other DTI measures. Therefore, information on each individuals’ AD (the 1st eigenvalue), RD (the average of the 2nd and 3rd eigenvalues), and MD was fed into FSL’s Randomize tool along with the mask based on our ROI analysis.

## Results

From the original total of 22 PTSD and 30 control adolescents, three controls were excluded because of image artifacts in T1-weighted anatomical scans. Further, two adolescents with PTSD were excluded because of image artifacts in the DTI dataset, resulting in a final sample of 20 adolescents with PTSD. From the remaining 27 controls, 20 subjects were group-wise matched on age and gender with the PTSD adolescents. Eventually, 40 participants (20 PTSD and 20 controls) were included. Of the 20 PTSD participants, 19 fulfilled all PTSD criteria on the ADIS, while one had sufficient PTSD symptoms, but with limited interference. Since earlier research showed that persons with subthreshold PTSD in many aspects resemble PTSD patients, we decided to include this patient [10].

The majority of participants was female (88 %, see Table 1). Of the participants with PTSD, 15 had comorbid anxiety disorders, most often more than one. Eight had a comorbid depressive disorder and one an oppositional defiant disorder (ODD). All controls and 16 adolescents with PTSD were drug and treatment naïve. Two adolescents with PTSD were on stable SSRI treatment and two used amphetamines (not on the day of scanning).

**Table 1:** Demographic and clinical characteristics of participants

	PTSD (*N* = 20)		CNTR (*N* = 20)		*p*
	Mean	SD	Mean	SD	
Gender (f : m)	17:3		18:2		
Age (in months)	198	24	185	19	0.06
FIQ	99	9	107	9	<0.01
*PDS* ^a^					
Pre/mid-pubertal	1		4		
Late pubertal	6		9		
Post-pubertal	10		5		
*TSCC* ^b^					
Anxiety	9.3		6.0		3.3
Depression	9.9		4.9		2.6
Anger	6.2		3.5		2.0
Post-traumatic stress	11.8		7.2		2.3
Dissociation	8.6		5.6		2.7
Sexual concerns	4.7		3.3		1.3

Because less than 20 % of the data in TSCC were missing, expectation maximization as regression method was used to calculate the scale scores

^a^Missing data: 2 in control group, 3 in PTSD group

^b^Three PTSD participants did not complete the questionnaire

The PTSD group had a significantly lower FIQ than controls [*F*(1, 38) = 8,14, *p* < 0.01] and more subjects in the post-pubertal phase (50 versus 25 %). As expected, there was a significant main effect of group on the TSCC scale scores [with and without controlling for age and FIQ; *F*(7,28) = 6,48, *p* < 0.01]. The PTSD group had significantly higher scores on all TSCC scales (all with *p* < 0.01).

### TBSS Analyses

**Fig. 1:**
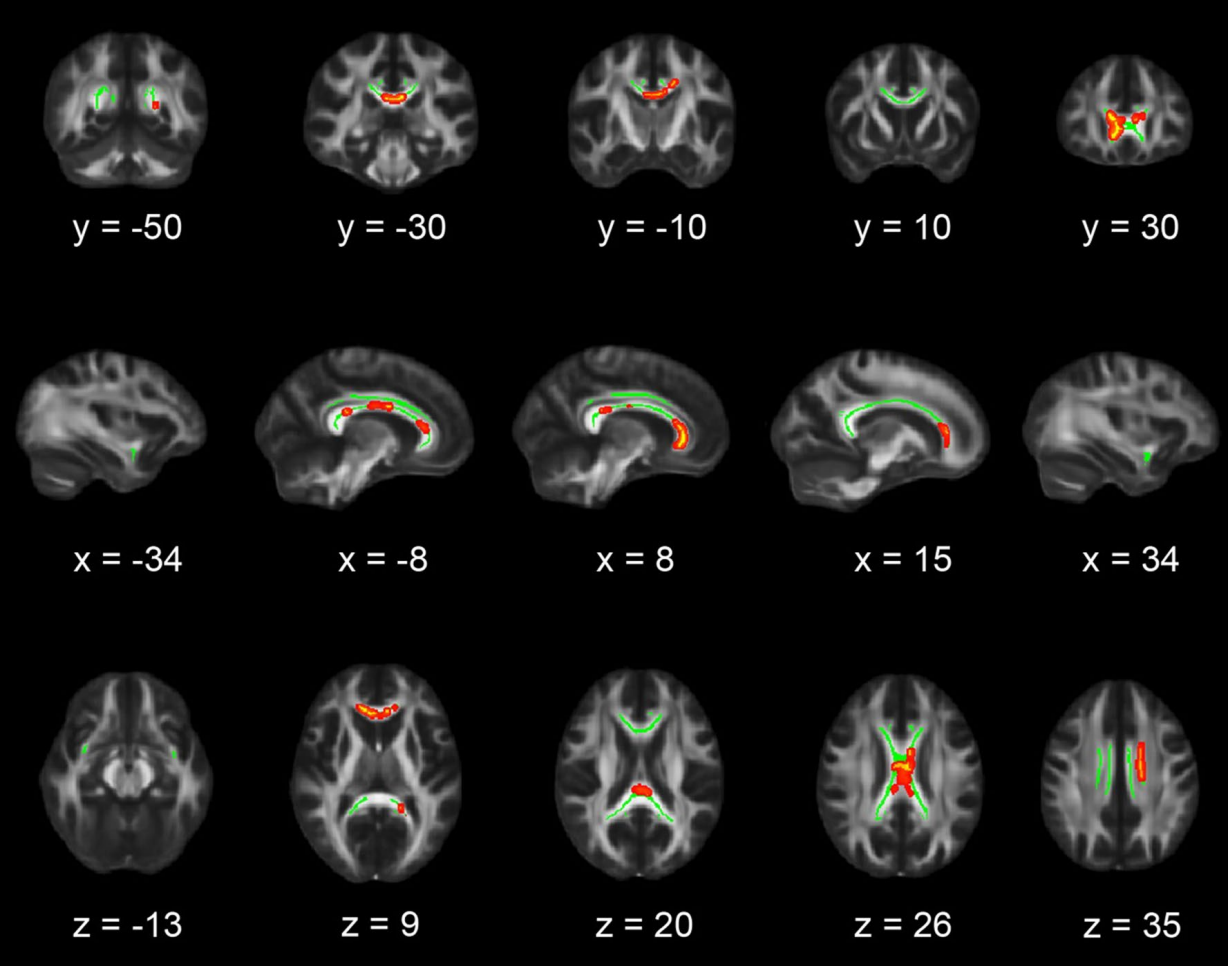
Region-of-interest analysis results. Coronal, sagittal and transversal axial sections of the white matter skeleton (*green*) superimposed on the FMRIB58_FA_1 mm standard brain (*gray*). Depicted in *yellow* are the regions in which FA values are significantly smaller in patients with PTSD compared to matched healthy controls. For better visibility, the results are thickened using the “tbss-fill” command (*red*). All TBSS results are corrected for multiple comparisons (*p* < 0.05, TFCE corrected), and the axial images are in radiological convention (the *right side* of the image corresponds with the left hemisphere of the brain and vice versa)

**Fig. 2:**
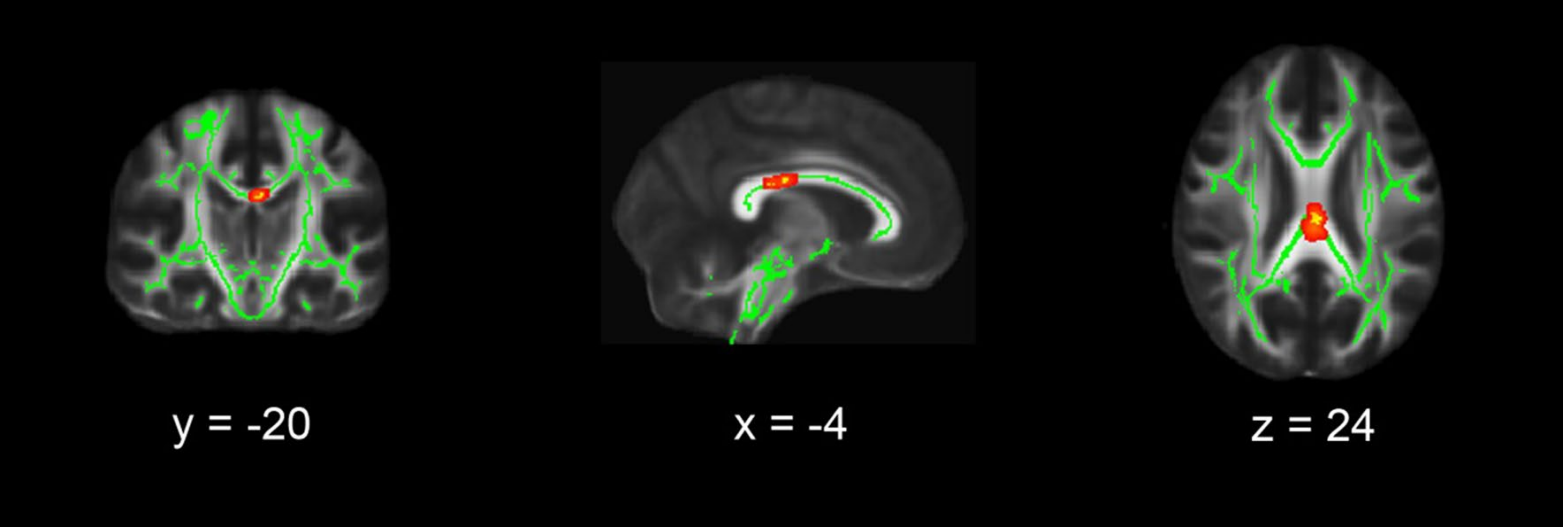
Whole brain TBSS results. Coronal, sagittal and transversal axial sections of the white matter skeleton (*green*) superimposed on the FMRIB58_FA_1 mm standard brain (*gray*). Depicted in *yellow* are the regions in which FA values are significantly smaller in patients with PTSD compared to matched healthy controls. For better visibility, the results are thickened using the “tbss-fill” command (*red*) All TBSS results are corrected for multiple comparisons (*p* < 0.075, TFCE corrected), and the axial images are in radiological convention (the *right side* of the image corresponds with the left hemisphere of the brain and vice versa)

ROI-based TBSS analysis showed that, in comparison with controls, the PTSD group had lower FA values in the genu, midbody and splenium of the CC (*p* < *0*.05, TFCE corrected) (Fig. 1). No FA differences were observed in the bilateral UF and cingulum. The exploratory whole brain TBSS revealed no significant lower FA values. When the threshold was lowered we found lower FA values in the body of the CC in the left hemisphere, adjacent to the splenium (*p* < 0.075, TFCE corrected; Fig. 2). No white matter tracts with lower FA values were found for controls versus PTSD.

**Fig. 3:**
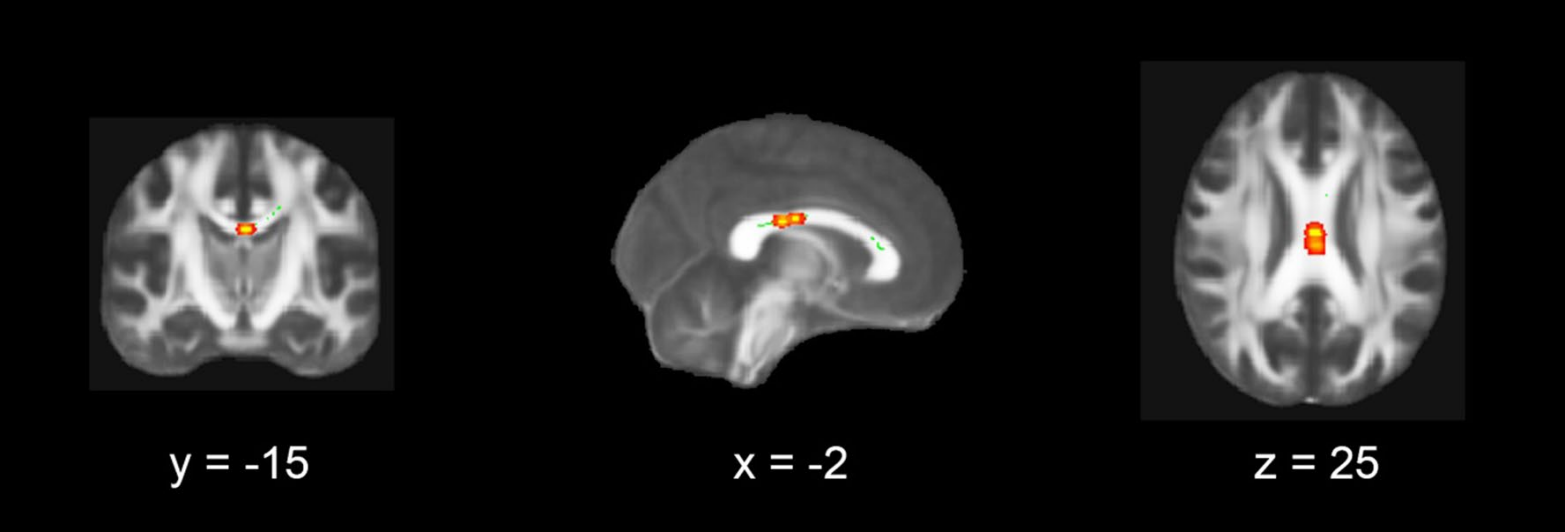
Voxel-wise correlation between TSCC anger subscale scores and FA values in adolescents with PTSD. Coronal, sagittal and transversal axial sections of the white matter skeleton (*green*) superimposed on the FMRIB58_FA_1 mm standard brain (*gray*). FA values in the *left* CC correlated negatively with TSCC anger subscale scores (*p* < 0.05) in the adolescents with PTSD (*yellow*). For better visibility, the results are thickened using the “tbss-fill” command (*red*). The axial images are in radiological convention (the *right side* of the image corresponds with the left hemisphere of the brain and vice versa)

Using a voxelwise correlation approach, we examined the association between the observed smaller FA values from the ROI analysis, and the TSCC total and subscale scores in the patients. We found a significant negative correlation between scores on the anger subscale of the TSCC and FA values in the left body of the CC (*p* < 0.05; uncorrected, Fig. 3.)

Post hoc analyses of the AD, RD and MD in the voxels that showed FA differences between groups revealed a significant increase (*p* < 0.05, TFCE corrected) of RD and MD in the PTSD group compared to controls. No significant differences were found between groups in AD. Omitting the one CSA participant who met all PTSD criteria except for interference did not change our findings. Excluding the two participants that were using medication from the analyses did not change the results either.

## Discussion

We examined white matter integrity in a sample of adolescents with CSA-related PTSD, using an ROI and an additional exploratory whole brain approach. We hypothesized reduced FA in a number of relevant white matter tracts: the CC, UF and cingulum. Compared to the control group, our adolescent PTSD group only showed decreased FA in areas of the CC, with additional DTI parameters suggesting demyelinization and dysmyelinization in these areas. We also found a significant correlation (uncorrected) between FA in the CC and Anger scores on the TSCC in the adolescents with CSA-related PTSD.

This study is the first to report on white matter integrity in a group of adolescents with CSA-related PTSD. The results of our study are in line with the findings of the recent DTI study by Jackowski and colleagues, who examined the CC in a group of children with PTSD following various forms of intrafamilial maltreatment, and also found reduced FA in several subregions of the CC [24]. Our findings are in line with recent reviews indicating that the most consisting finding in youth with psychotrauma is structural abnormalities of the CC, in contrast to the reduction of hippocampal volume typically reported in adults with PTSD [5, 40].

The CC is known to change throughout life, but most dramatically during childhood and adolescence [2, 30]. These developmental changes in the CC are the consequence of varying degrees of axonal myelinization, redirection, and pruning, reflecting a permanent adjustment and fine-tuning of fibers connecting homologous cortical areas. The general trend during adolescence is toward increasing FA and decreasing MD [43]. This CC maturation parallels puberty development suggesting gonadal hormonal influences [2]. For this reason, we included PDS scores as regressor. However, we must acknowledge that a simple linear regression of pubertal stage and total brain volume may still not sufficiently account for the results as they are known not to be linear across adolescence.

Early traumatization is likely to have a major influence on the integrity of the CC, as the processes of myelinization and selective pruning are typically influenced by stress hormones [52, 54]. Of importance, the smaller FA values we found in the CC of the PTSD group were due to increases in RD and MD, known to reflect demyelinization (less development of the myelin sheet) and dysmyelinization (aberrant development of the myelin sheet), linking the abnormalities of the CC integrity to the possible influence of stress hormones. Supporting this possible association, a recent study found that in rhesus monkeys exposed to early maternal abuse, cortisol levels at the time of abuse correlated with abnormalities in white matter connectivity in the CC, brain stem and other brain areas in adolescence [20]. Our results are in line with the study of Teicher et al. who, comparing abuse and neglect, found that sexual abuse was the strongest factor influencing CC size in girls [53].

Recent topographic research on the CC is beginning to map the different regions of the CC and their connections. Apart from frontal connections, the body of the CC also has connections with subcortical nuclei [21]. Changes in the midbody of the CC in children and adolescents who experienced psychological trauma could be related to disturbances in connectivity with limbic subcortical nuclei, resulting from or underlying the disturbances in emotion regulation.

We found a negative association in the adolescent PTSD group between FA in the CC and the TSCC Anger subscale. This in contrast to the result of the small DTI study in socioemotional deprived children with PTSD [14] in which correlations of FA measures in the CC were found with total anxiety scores, panic scores and separation anxiety scores. This may be due to methodological differences as well as the populations studied. A preclinical study in male non-human primates examined the effects of early life stress on hippocampal volume and CC development and found a significant inverse relationship between CC mid-sagittal area in adult monkeys and the response toward an intruder which typically consists of a mixture of aggressive and anxious behavior [23]. In a recent DTI study in male adolescents with conduct disorder, Zhang et al. report increased structural connectivity in the genu and body of the CC [61]. Impulsivity correlated positive with WM integrity, which is the opposite pattern of what we found in our study. This suggests that different pathophysiological mechanisms are involved, which is in accordance with the putative mechanisms described in the literature. For instance, Raine et al. hypothesize that structural abnormalities in the CC are a consequence of an early arrest of the normal neurodevelopmental process of axonal pruning, while the abnormalities in myelinization following CSA may be more linked to detrimental stress hormone influences [39].

We believe the relatively homogeneous sample and the state-of-the-art DTI approaches are strengths of our study, although several potential limitations should be taken into account. While we know that gender influences brain development and the reaction to psychological trauma, we included gender as a regressor, but could not further explore this issue because our participants are mainly girls. Full scale IQ measures differed significantly between the PTSD group and controls. Several studies report a negative effect of ELS on cognitive function [13, 37]. In this respect intellectual ability in the PTSD group and the control group might originally have been more equal. The New Zealand longitudinal birth cohort study [28] instead points to IQ as a risk factor for the development of PTSD. In the discussion about lower IQ being a consequence or a predictor of PTSD, it is suggested that trauma severity overrules IQ as a predictor [33] which could be the case in our study where all included adolescents fulfilled symptom criteria for PTSD diagnosis although this was not an inclusion criterion, but here too results are inconclusive [6]. Navas-Sanchez et al. [35] found a positive correlation between IQ and FA in the CC in math gifted adolescents compared to controls matched for age and academic level. Other studies report about correlations, mostly positive, of cognitive function with FA in several WM tracts [17, 42]. To decrease the potential influence of IQ on the white matter integrity differences we included IQ as a covariate in our analyses, but clearly further research is needed to unravel the exact relationship between childhood adversity, IQ and WM integrity in adolescence.

The normal increase of FA in the CC during adolescence is related to pubertal development. Therefore, the PDS score is chosen as nuisance regressor instead of age. The PTSD group was older and more advanced in pubertal stage. Because of the expected increase, the decrease in FA found in the PTSD group cannot be a consequence of normal (pubertal) development.

Two adolescents with PTSD were on stable SSRI treatment. Omitting these two participants from our analyses did not have any effect on the results. To our knowledge no influence of SSRIs on the CC is reported [9].

Patients were selected based on the presence of CSA and all except one, who fulfilled PTSD criteria except interference, showed CSA-related PTSD. Hence, we cannot differentiate whether the neuroimaging results were a consequence of exposure to trauma or the (development of) PTSD pathology or reflect an underlying vulnerability. Previous research in twins discordant for combat exposure suggests that anatomical abnormalities may indeed represent pre-existing vulnerability factors [16]. The ideal cross-sectional design to disentangle the effects of exposure, psychopathology and resilience would have incorporated a CSA group with psychopathology, a CSA group without psychopathology and a non-exposed, healthy control group [55]. Furthermore, we could not assess the influence of timing and duration of the CSA in our study, which is thought to be highly relevant in children and youth.

About one-third of the subjects also reported physical abuse, but as we did not assess experiences of other forms of psychotrauma we could have missed other prevalent traumatic experiences, like emotional maltreatment, which might be associated with the presence of DTI abnormalities in our sample.

In conclusion, our DTI findings in this sample of adolescents with CSA-related PTSD point at the involvement of the CC in brain alterations associated with juvenile sexual psychotrauma, and together with recent animal data can be taken to point at the influence of stress hormone levels on CC integrity. Clearly, longitudinal studies till mid-adulthood are needed to further elucidate the role of altered CC white matter integrity in the biopsychological consequences of early traumatization and to examine its malleability.
